# Fruit softening: evidence for rhamnogalacturonan lyase action *in vivo* in ripe fruit cell walls

**DOI:** 10.1093/aob/mcad197

**Published:** 2024-01-05

**Authors:** Thurayya Z S Al-Hinai, C Logan Mackay, Stephen C Fry

**Affiliations:** The Edinburgh Cell Wall Group, Institute of Molecular Plant Sciences, The University of Edinburgh, Daniel Rutherford Building, The King’s Buildings, Max Born Crescent, Edinburgh EH9 3BF, UK; EastCHEM School of Chemistry, The University of Edinburgh, The King’s Buildings, Edinburgh EH9 3FJ, UK; The Edinburgh Cell Wall Group, Institute of Molecular Plant Sciences, The University of Edinburgh, Daniel Rutherford Building, The King’s Buildings, Max Born Crescent, Edinburgh EH9 3BF, UK

**Keywords:** Cell wall, Driselase, fruit softening, high-voltage paper electrophoresis, rhamnogalacturonan-I, rhamnogalacturonan lyase, pectate lyase

## Abstract

**Background and Aims:**

The softening of ripening fruit involves partial depolymerization of cell-wall pectin by three types of reaction: enzymic hydrolysis, enzymic elimination (lyase-catalysed) and non-enzymic oxidative scission. Two known lyase activities are pectate lyase and rhamnogalacturonan lyase (RGL), potentially causing mid-chain cleavage of homogalacturonan and rhamnogalacturonan-I (RG-I) domains of pectin respectively. However, the important biological question of whether RGL exhibits action *in vivo* had not been tested.

**Methods:**

We developed a method for specifically and sensitively detecting *in-vivo* RGL products, based on Driselase digestion of cell walls and detection of a characteristic unsaturated ‘fingerprint’ product (tetrasaccharide) of RGL action.

**Key Results:**

In model experiments, potato RG-I that had been partially cleaved *in vitro* by commercial RGL was digested by Driselase, releasing an unsaturated tetrasaccharide (‘ΔUA-Rha-GalA-Rha’), taken as diagnostic of RGL action. This highly acidic fingerprint compound was separated from monosaccharides (galacturonate, galactose, rhamnose, etc.) by electrophoresis at pH 2, then separated from ΔUA-GalA (the fingerprint of pectate lyase action) by thin-layer chromatography. The ‘ΔUA-Rha-GalA-Rha’ was confirmed as 4-deoxy-β-l-*threo*-hex-4-enopyranuronosyl-(1→2)-l-rhamnosyl-(1→4)-d-galacturonosyl-(1→2)-l-rhamnose by mass spectrometry and acid hydrolysis. Driselase digestion of cell walls from diverse ripe fruits [date, sea buckthorn, cranberry, yew (arils), mango, plum, blackberry, apple, pear and strawberry] yielded the same fingerprint compound, demonstrating that RGL had been acting *in vivo* in these fruits prior to harvest. The ‘fingerprint’ : (galacturonate + rhamnose) ratio in digests from ripe dates was approximately 1 : 72 (mol/mol), indicating that ~1.4 % of the backbone Rha→GalA bonds in endogenous RG-I had been cleaved by *in-vivo* RGL action.

**Conclusions:**

The results provide the first demonstration that RGL, previously known from studies of fruit gene expression, proteomic studies and *in-vitro* enzyme activity, exhibits enzyme action in the walls of soft fruits and may thus be proposed to contribute to fruit softening.

## INTRODUCTION

Cell-wall modifications during ripening can be non-enzymic and/or enzymic. Non-enzymic modifications include pectic polysaccharide depolymerization by reactive oxygen species (especially the hydroxyl radical ^•^OH; Dumville and Fry, 2000; Airianah *et al.*, 2016). Wall-modifying enzymes studied in relation to fruit softening include cellulases (Dong *et al.*, 2018), xyloglucan endotransglucosylase/hydrolases (XTHs; Saladié *et al.*, 2006; Miedes and Lorences, 2009), endo-polygalacturonases (EPGs; Wu *et al.*, 1993; Asif and Nath, 2005; Quesada *et al.*, 2009), pectate lyases (PLs; Marín-Rodríguez *et al.*, 2003; Dong *et al.*, 2018; Al-Hinai *et al.*, 2021), rhamnogalacturonan-I lyases (RGLs; Ochoa-Jiménez *et al.*, 2018*a*, *b*; Méndez-Yañez *et al.*, 2020), pectin methylesterases (PMEs; Tieman *et al.*, 1992; Phan *et al.*, 2007), β-galactosidases (Smith *et al.*, 2002; Paniagua *et al.*, 2016), α-arabinosidases (Sozzi *et al.*, 2002; Brummell *et al.*, 2004; Tateishi, 2008) and exo-polygalacturonases (Bartley, 1978; Yang *et al.*, 2018).

### Pectin modification during fruit softening

Pectin has three major covalently interlinked domains: homogalacturonan (HG), rhamnogalacturonan-I (RG-I) and RG-II, which are synthesized in Golgi vesicles (Caffall and Mohnen, 2009; Fry, 2010).

Homogalacturonan (‘pectate’) domains often constitute more that 60 % of the pectin (Caffall and Mohnen, 2009) and consist of unbranched chains of anionic (1→4)-α-d-galacturonate (GalA) residues plus neutral blocks of methyl-esterified (1→4)-α-GalA residues. RG-I domains constitute up to 35 % of pectin (Mohnen, 2008) and have a backbone of repeating disaccharide units of -(1→4)-α-d-GalA-(1→2)-α-l-Rha- (where Rha = rhamnose), with neutral side-chains of β-galactose (Gal) and/or α-arabinose (Ara) usually attached to ~50 % of the Rha residues at their *O*-4 position. There is considerable inter-specific variation in the structure of RG-I (Kaczmarska *et al.*, 2022). The GalA residues of the RG-I backbone are not methyl-esterified; however, they can be *O*-acetylated at C-2 and/or C-3 (Ishii, 1997; Perrone *et al.*, 2002; Yapo *et al.*, 2007). In some species such as sugarbeet, the backbone of RG-I includes some glucuronate (GlcA) residues (Renard and Jarvis, 1999). RG-II consists of seven to ten (1→4)-α-d-GalA residues as a backbone to which six different side-chains are attached, making a highly complicated structure, usually occurring as a boron-bridged dimer (Kobayashi *et al.*, 1996; Begum and Fry, 2023).

Enzymes that may cleave pectin domains in mid-chain, thus potentially contributing to wall loosening during fruit ripening, are EPGs, PLs and RGLs (Wang *et al.*, 2018). In addition, exo-polygalacturonases (Bartley, 1978; Yang *et al.*, 2018) attack homogalacturonan but not by mid-chain cleavage. Roles of β-d-galactosidase and α-l-arabinofuranosidase in the degradation of RG-I side-chains have also been suggested (Goulao *et al.*, 2007).

Data are available on the transcription of genes encoding the pectin endo-cleaving enzymes EPG, PL and RGL in ripening fruit (Tucker and Grierson, 1982; Molina-Hidalgo *et al.*, 2013; Uluisik *et al.*, 2016; Yang *et al.*, 2017); on the translation of the corresponding mRNAs to generate the enzymic proteins (Brummell and Harpster, 2001; Dautt-Castro *et al.*, 2015; Uluisik *et al.*, 2016); and on the activity of the enzymes as assayed *in vitro* in plant extracts (Ochoa-Jiménez *et al.*, 2018*a*; Wang *et al.*, 2018; Méndez-Yañez *et al.*, 2020; Uluisik and Seymour, 2020; Zhang *et al.*, 2020). Together, these observations lend support to the idea that EPG, PL and RGL serve roles in fruit development. However, there is little direct evidence for the action of the enzymes as demonstrated by observed chemical changes in the substrate polysaccharides *in vivo* (Al-Hinai *et al.*, 2021; Seymour, 2021), a topic which is the focus of the present paper.

### RG-I-modifying enzymes

**Fig. 1.**
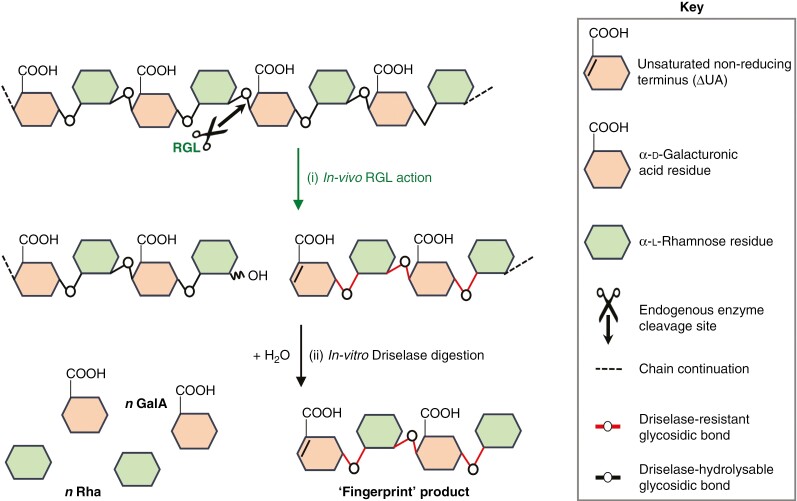
Rhamnogalacturonan lyase (RGL) action and an experimental strategy to demonstrate it *in vivo*. Reaction (i): RGL attacks the α-(1→4)-glycosidic bond between Rha and GalA of the RG-I backbone by β-elimination (potentially *in vivo*), producing a new Rha-reducing terminus and a new unsaturated non-reducing (ΔUA) terminus. Reaction (ii): *in-vitro* digestion of RGL products with Driselase cleaves the whole chain of RG-I to GalA and Rha monomers (plus Gal and Ara from the side-chains if present; not shown) plus the tetramer, ΔUA–Rha–GalA–Rha, the unique RGL action fingerprint characterized here.

Here we focus on the enzymic endo-cleavage of RG-I. RGL cleaves the α-(1→4)-glycosidic bonds between Rha and GalA of the RG-I backbone via β-elimination, producing a new Rha reducing end and a new unsaturated uronic acid (ΔUA) non-reducing end [Fig. 1, reaction (i)] (McKie *et al.*, 2001; McDonough *et al.*, 2004; Ochoa-Jiménez *et al.*, 2018*a*). We use the term ‘ΔUA’ rather than ‘ΔGalA’ because it is identical to the product which would be generated from GlcA.

RGL (first erroneously referred to as ‘rhamnogalacturonase B’) was first purified from the fungus *Aspergillus aculeatus* and tested on RG-I (Mutter *et al.*, 1996). *Aspergillus* RGL is more active on RG-I molecules that carry neutral Gal side-chains on their backbone Rha residues, and less active on acetylesterified RG-I (Mutter *et al.*, 1998). Bacterial RGLs have also been reported, and Wang *et al.* (2022) reported a difference in the substrate preference of two bacterial RGLs, one showing a higher activity against RG-I with galactan and arabinan side-chains while the presence of such side-chains inhibited the activity of the other isoform. It is possible that plant RGLs do not attack the backbone of RG-I efficiently in the vicinity of the Ara- and/or Gal-rich side-chains or adjacent to the GlcA residues if any – a question that remains to be answered. An RGL gene expressed in swollen receptacles (‘fruit’) of ripening strawberry was reported, and silencing this gene resulted in a more intact middle lamella and firmer berries (Molina-Hidalgo *et al.*, 2013). The gene (*Solyc11g011300*) encoding a putative RGL was highly expressed in the flowers and red-ripe fruits of tomato (Ochoa-Jiménez *et al.*, 2018*a*, *b*), though the enzymic activity of the encoded protein was not tested. In addition, Dautt-Castro *et al.* (2015) reported a 5-fold increase in the expression of an RGL gene in ripe mango compared with mature green fruit. Extractable RGL activity was reported in tomato (Ochoa-Jiménez *et al.*, 2018*a*) and Chilean strawberry fruits (Méndez-Yañez *et al.*, 2020) but could not be found in cotton cotyledons (Naran *et al.*, 2007).

Naran *et al*. (2007) reported RGL activity *in situ* in the intercellular spaces of expanding cotton cotyledons when these were infiltrated with RG-I oligosaccharides (tetradeca- to octadecasaccharides, mimicking the much larger backbone of RG-I) fluorescently labelled with 8-aminopyrene-1,3,6-trisulphonate. Smaller fluorescent products were produced with electrophoretic properties that implied RGL rather than hydrolase activity. The RGL activity measured by Naran *et al.* (2007) peaked during the period of most rapid cotyledon expansion and decreased with diminishing growth rate. These observations show that plant tissue can exhibit *in-vivo* RGL action on freely soluble exogenous RG-I oligosaccharides but do not prove *in-vivo* action on endogenous RG-I polysaccharides that are insoluble components of the cell-wall architecture.

Thus, no evidence was yet available to prove RGL action on endogenous RG-I in the walls of living tissues – for example, cotton cotyledons that had not been fed exogenous RG-I oligosaccharides did not show any recognizable RGL products (Naran *et al.*, 2007). The present work sought evidence for such action in ripening fruits.

A second endo-acting enzyme activity that could potentially cleave the RG-I backbone is rhamnogalacturonase (RGase; EC 3.2.1.171). This enzyme is well documented in certain fungi, such as the phytopathogen *Aspergillus aculeatus*; it catalyses endo-hydrolysis of an α-d-GalA-(1→2)-α-l-Rha glycosidic bond in the backbone of RG-I (Azadi *et al.*, 1995), creating a product with an Rha non-reducing terminus. Similar activity has been suggested to occur in fruits of tomato, apple and grape (Gross *et al.*, 1995), in carrot roots (Stratilová *et al.*, 1998), and in germinating *Orobranche* seeds (Veronesi *et al.*, 2007), but was not detected in the study by Naran *et al.* (2007). Certainly, RGase activity is less well documented in plants than in fungi. This proposed enzyme activity would not generate the ΔUA-containing ‘fingerprint’ shown in Fig. 1 as it does not introduce a double-bond. Thus, the method proposed in the present paper is expected to be specific for detecting RGL action.

The ^•^OH radical can also cleave polysaccharides (presumably including RG-I) in mid-chain, forming diverse new termini. Based on studies of model substrates (Schuchmann & von Sonntag, 1978; von Sonntag, 1987; Lindsay & Fry, 2007; Airianah *et al.*, 2016), the newly generated termini in ^•^OH-treated RG-I are expected to include GalA, Rha, l-galactonic acid (l-GalO), l-GalO-lactone, rhamnonic acid and rhamnonolactone (at the ‘reducing’ terminus), and Rha, GalA and the corresponding glycosulose residues (rhamnos-2-ulose and urono-4-ulose) (at the non-reducing end). These products do not contain C=C double bonds as found in ΔUA, and thus again the ΔUA-containing ‘fingerprint’ shown in Fig. 1 is expected to be specific for detecting RGL action.

Following up our recent strategy for demonstrating PL action by detection of a ‘fingerprint’ product in various fruits (Al-Hinai *et al.*, 2021), the present study uses a similar approach to detect a unique RGL fingerprint product in fruits. The proposed strategy involves digesting fruit cell walls with Driselase, which is expected to release monosaccharides plus small oligosaccharide(s) including an RGL action fingerprint (of the type ΔUA-Rha-[GalA-Rha]_*n*_), indicated in Fig. 1 reaction (ii). High-voltage paper electrophoresis (HVPE) is then used to sort the Driselase products based on their charge : mass ratio, which should separate all products containing a ΔUA residue (which has a particularly low *p*K_a_; Kohn and Kováč, 1978) from the bulk (saturated) products.

## MATERIALS AND METHODS

### Materials

Date (*Phoenix dactylifera* ‘Khalas’) and mango (*Mangifera indica*) fruits were collected from randomly selected trees in a private field in Oman in June 2018. The samples were stored at −80 °C. Pear (*Pyrus communis* ‘Conference’), rowan (*Sorbus aucuparia*), apple (*Malus pumila* ‘Bramley’), yew aril (*Taxus baccata*), blackberry (*Rubus fruticosus*), plum (*Prunus domestica*) and raspberry (*Rubus idaeus*) fruits were collected from private gardens in Edinburgh, UK. Sea buckthorn (*Hippophae rhamnoides*) fruit was wild-collected in East Lothian, UK. Cranberry (*Vaccinium macrocarpon*) was from a supermarket. All fruits tested were ripe, as judged by softness and colour. In the case of the larger fruits (apples, pears, plums), at least three individual fruit were pooled for analysis.

RGL (from *Dickeya dadantii*, Nzytech; https://www.nzytech.com) was supplied in 35 mm HEPES (Na^+^), pH 7.5, 750 mm NaCl, 200 mm imidazole, 3.5 mm CaCl_2_ and 25 % (v/v) glycerol. Driselase (from a basidiomycete, Sigma-Aldrich; https://www.sigmaaldrich.com) was de-salted by ammonium sulphate precipitation and gel-permeation chromatography (Fry, 2000), dried and re-dissolved in pyridine/acetic acid/H_2_O (1 : 1 : 98 by vol., containing 0.5 % chlorobutanol). Potato RG-I was from Megazyme (https://www.megazyme.com). Aluminium-backed F254 silica-gel thin-layer chromatography (TLC) plates (1.05554.0001) were from Merck (https://www.merckgroup.com).

### Preparation of alcohol-insoluble residue (AIR) from fruits

AIR from each fruit sample was prepared as the source of cell walls. Fruit skin and seeds were removed when possible. Then, 9 g of fresh fruit was homogenized in 75 % ethanol (final concentration, considering the fruit fresh weight as water) containing 5 % formic acid. The homogenate was incubated on a wheel for 16 h and then centrifuged at 3220 *g* for 5 min. The pellet was washed with neutral 75 % ethanol on a wheel for 1 h, then re-centrifuged; the supernatant was discarded. Prior to digestion with Driselase, the AIR (~0.3 g dry weight) was de-esterified in 10 mL aqueous 0.2 m Na_2_CO_3_ with shaking at 4 °C for 16 h. Acetic acid was then added to bring the pH to <5. Pure ethanol was added to make a final concentration of 75 % and the tube was incubated on a wheel for 1 h. The mixture was re-centrifuged, the supernatant discarded and the pellet washed in 75 % ethanol three times on a wheel for 1 h each as above. The pellet was then washed twice in acetone each for 1 h on a wheel. The final pellet (AIR) was dried and stored at room temperature.

### RG-I digestion

Potato RG-I was freed of any ester groups (e.g. *O*-acetyl esters) by incubation in 60 mm NaOH for 1 h at 20 °C, then neutralized with acetic acid, dialysed against 0.5 % chlorobutanol, freeze-dried and washed three times in 65 % ethanol to remove the last traces of oligosaccharides. The remaining insoluble material was re-dried and used as purified RG-I.

A reaction mixture containing 2.9 mg mL^–1^ RG-I, 14.7 mm lutidine (acetate) buffer (pH 6) and 0.03 mg mL^–1^ of commercial RGL was incubated at 37 °C on a shaker for 16 h. The reaction was stopped by addition of 0.2 volumes of formic acid. The products were dried and digested with Driselase to release the smallest unsaturated products, which were then resolved by HVPE.

### Driselase digestion

Driselase digestion was conducted on RG-I, RGL-pretreated RG-I and natural fruit AIR. In the case of AIR, the reaction contained 30 mg AIR plus 3 mL of 0.5 mg mL^–1^ de-salted Driselase, buffered with pyridine/acetic acid/H_2_O (1 : 1 : 98 by vol., pH 4.7) containing 0.5 % chlorobutanol. The reaction mixture was incubated on a shaker at 37 °C, typically for 3 d, then the reaction was stopped by the addition of 0.2 volumes of formic acid and the products were dried in a SpeedVac. The Driselase digestion products of 30 mg AIR were re-dissolved in 1.2 mL H_2_O and stored frozen at −20 °C. The digestion products of 3 mg of commercial RG-I were dissolved in 600 µL H_2_O.

### High-voltage paper electrophoresis

Driselase digestion products of 30 mg AIR or 3 mg RG-I were loaded as a 6-cm streak on Whatman No. 3 paper. Electrophoresis was conducted at pH 2.0 in a volatile buffer [formic acid/acetic acid/water (1 : 3.5 : 35.5, by vol.)] at 3 kV for 4 h. The apparatus and methods are described by Fry (2020). A small part of the paper (the fringe of the sample streak plus the whole neighbouring ΔUA–GalA_*n*_ marker track) was stained with AgNO_3_ (Fry, 2000). Unsaturated oligogalacturonides were eluted from specific 1-cm zones of the unstained part of the electrophoretogram in 75 % ethanol, dried and re-dissolved in 50 µL of H_2_O.

### Thin-layer chromatography

Samples eluted from preparative paper electrophoretograms were loaded on aluminium-backed F254 silica-gel TLC plates as an 8-mm streak of 2.5 µL. The plate was run in butan-1-ol/acetic acid/water (BAW, 2 : 1 : 1) for 7 h and then dried. After drying, the plate was stained in thymol solution (0.5 % w/v thymol and 5 % v/v H_2_SO_4_ in ethanol) followed by re-drying and then heating in an oven at 105 °C for 5 min, and scanned.

### Mass spectrometry

Samples were prepared for electrospray mass spectrometry (MS) analysis at a concentration of ~10 µm in acetonitrile/water (1 : 1). Analysis was performed on a 12-T SolariX 2XR Fourier-transform ion cyclotron resonance (FT-ICR) mass spectrometer (Bruker Daltonics) operating in either positive or negative mode. Each spectrum was the sum of 20 scans, with a data set size of 2 million words. Fragmentation was performed by collision-induced dissociation (CID) with argon as a neutral gas. The collision voltage was 10 V. Data interpretation was achieved with DataAnalysis 5.0 (Bruker Daltonics).

## RESULTS

### Products formed by Driselase digestion of potato RG-I

**Fig. 2.**
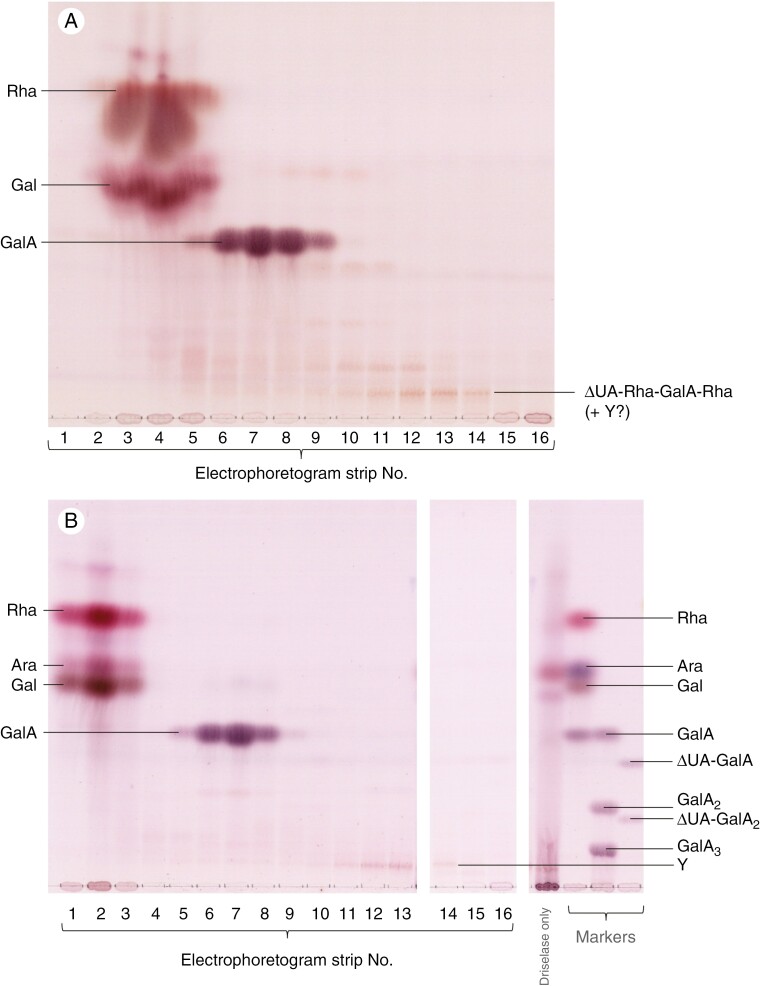
Driselase digestion products of commercial potato RG-I pre-treated, or not, with commercial RGL. (A) With RGL treatment and (B) without. In (A), de-esterified potato RG-I (2.9 mg mL^–1^) was incubated with 0.03 mg mL^–1^ commercial RGL in 14.7 mm lutidine (acetate) buffer, pH 6.0, at 37 °C for 16 h. The reaction was stopped with formic acid and the dried products were further digested with 0.5 mg mL^–1^ Driselase. This digestion was then stopped with formic acid and products were electrophoresed at pH 2 and 3 kV for 4 h. The electrophoretogram was then cut into 16 strips, each of which was eluted in 75 % ethanol and run by TLC (shown here). In (B), the RGL pre-treatment was omitted. ‘Y’, unidentified contaminant.

The ability of crude Driselase (two batches, E4 and E5) to digest various acidic cell-wall constituents was tested (Supplementary Data Fig. S1). Each digest contained free Glc (a contaminant of crude Driselase), and enzyme E5 had an additional, unidentified, contaminant. In future work with de-salted Driselase (e.g. Fig. 2), these contaminating sugars were not observed. All the acidic wall constituents tested yielded free GalA. In addition, RG-I gave the expected neutral sugars (Gal and Rha; little Ara was present in this commercial RG-I preparation). A mixture of ΔUA-GalA_1_, ΔUA-GalA_2_ and ΔUA-GalA_3_ (obtained as in Al-Hinai *et al.*, 2021) was digested to the smallest Driselase-stable product, ΔUA-GalA_1_. RG-II is not digested by Driselase (Chormova *et al.*, 2014). Pure GalA was not affected (e.g. oxidized) by Driselase under these conditions. Thus, Driselase was highly effective at digesting commercial RG-I, and the products appeared to be stable, as required in our subsequent experiments.

### Products formed by action of commercial RGL on pure RG-I in vitro followed by Driselase digestion

The *in-vitro* digestion of potato RG-I with commercial RGL followed by Driselase (Fig. 2A) revealed the expected neutral sugars (Gal and Rha, present in electrophoresis fractions 2–5), the acidic monosaccharide GalA (in fractions 5–9) and an interesting acidic oligosaccharide centred on fractions 11–14. The last of these was fast-migrating on electrophoresis, suggesting the presence of a low-*p*K_a_ residue such as ΔUA (Kohn and Kováč, 1978), but slow-migrating on TLC (Fig. 2A), suggesting a relatively large oligosaccharide. This was suspected to be the RGL ‘fingerprint’ product envisaged in Fig. 1.

The suspected fingerprint product was not visible in Driselase digests of RGL-untreated RG-I (Supplementary Data Fig. S1; Fig. 2B), confirming that the product observed in Fig. 2A was a result of RGL action and was neither a Driselase autolysis product nor due to a component already present in the RG-I prior to the RGL treatment. A minor spot (‘Y’) seen in Fig. 2B was present in crude Driselase run alone and was shown by MS *not* to be identical to the fingerprint product seen in Fig. 2A. It is likely that some ‘Y’ is also present in Fig. 2A.

### Mass spectrometry of the proposed RGL ‘fingerprint’ product

**Fig. 3.**
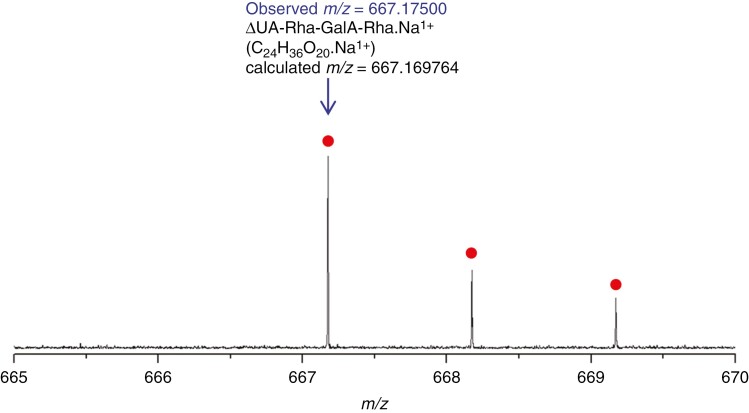
Mass spectrometry of the putative RGL fingerprint product obtained by digestion of commercial RG-I with commercial RGL followed by Driselase. Positive-mode ESI FT–ICR mass spectrum of sample from pooled electrophoresis fractions 11–14 (purified on a preparative TLC like that shown in Fig. 2A). Labelling in blue = observed; labelling in black = deduced. The mass error of the main peak is 8 ppm. Red dots indicate the same molecule with one or two naturally occurring ^13^C atoms.

The identity of the putative RGL fingerprint product, purified from a preparative paper electrophoretogram and eluted from a preparative TLC (run as in Fig. 2A), was supported by positive-mode MS. A peak was observed at *m*/*z* 667.17 500 (Fig. 3), corresponding to the sodium adduct of the tetrasaccharide ∆UA-Rha-GalA-Rha (C_24_H_36_O_20_.Na^+^; calculated mass 667.169 764, i.e. 8 ppm error), suggesting that the structure is indeed the non-reducing end of RG-I after attack by RGL followed by Driselase. Figure 3 shows small additional peaks due to the natural abundance of ^13^C.

The product has one ΔUA residue presumed to be at the non-reducing end. Since the backbone of RG-I consists of a GalA-Rha repeat unit, the findings indicate that the oligomeric RGL product was terminated by Rha (rather than GalA) at the reducing end, as also observed by Schols *et al.* (1990). The enzyme activity in Driselase responsible for releasing the fingerprint product from RGL-pre-treated RG-I is thus an endo-rhamnosidase.

### RGL fingerprint in Driselase digestion products from fruit cell walls: rowan as an example

Based on knowledge gained from the treatment of RG-I with RGL *in vitro*, a protocol to detect RGL action products *in vivo* was developed. Driselase digestion of de-esterified fruit cell walls (AIR) would be expected to cleave any RGL action products, even large ones such as ΔUA-(Rha-GalA)_*n*_ [with Ara- and/or Gal-rich side-chains] where *n* might be in the order of 10–100, to release GalA, Rha, Gal, Ara and the tetrasaccharide ΔUA-Rha-GalA-Rha. Paper electrophoresis was used to separate the highly acidic tetrasaccharide from all saturated sugars. TLC then resolved and visualized RGL products, providing evidence for RGL action *in vivo*. Driselase would also digest homogalacturonan [yielding GalA and potentially also ΔUA-GalA if previously attacked *in vivo* by PL (Al-Hinai *et al.*, 2021)], hemicelluloses (yielding monosaccharides, isoprimeverose and xylobiose), and a proportion of the cellulose (yielding glucose).

**Fig. 4.**
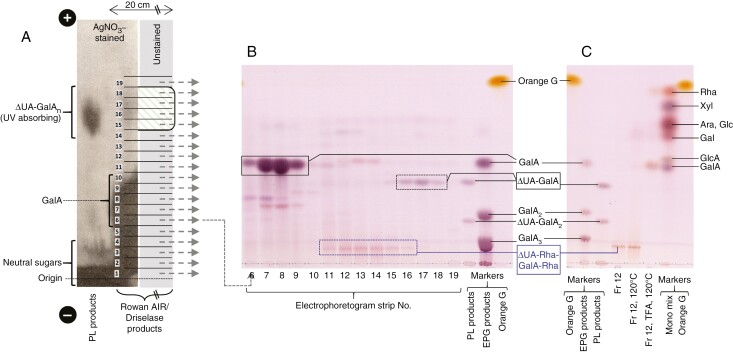
Detecting RGL (and pectate lyase) fingerprint products in digests of rowan berry cell walls. Rowan berry AIR (25 mg) was digested in 0.05 % de-salted Driselase. (A) Preparative HVPE: the products were loaded as a 20-cm streak on Whatman No. 3 paper and electrophoresed at pH 2.0 (3.0 kV for 4 h). The left-hand fringe of the 20-cm loading plus the adjacent markers (PL products generated *in vitro* from homogalacturonan) on the far left were stained with AgNO_3_. The major portion of the paper, only part of which is represented (in grey), was not stained; green hatching indicates a UV-absorbing band, mainly attributable to ΔUA-GalA. The whole unstained portion was cut into 19 1-cm strips and products were eluted. (B) Eluates from strips 6–19 were run by TLC alongside marker mixtures [PL products as in (A); EPG products, saturated oligogalacturonides; and Orange G], and stained with thymol/H_2_SO_4_. Blue box = putative RGL fingerprint product (tetrasaccharide). (C) The putative fingerprint compound seen in fraction 12 (Fr12) was re-run by TLC after no further treatment and after treatment at 120 °C for 1 h at neutral pH or in 2 m TFA (trifluoroacetic acid). The TFA was then removed by drying in a SpeedVac. Mono mix = monosaccharide marker mixture.

An example of the methodology is shown in Fig. 4. Paper electrophoresis (pH 2.0) of the products obtained by Driselase digestion of cell walls from rowan berries produced a heavy spot of neutral sugars, a heavy monomeric GalA spot and a faster-migrating, UV-absorbing spot indicating the presence of highly acidic, unsaturated products (Fig. 4A). Strips of the electrophoretogram were eluted and the compounds present were analysed by TLC (Fig. 4B right). The neutral fractions (strips 1–2; not shown) gave a range of neutral sugars (including isoprimeverose, Gal, glucose and Rha). Fractions 6–9, which had co-electrophoresed with GalA, were confirmed by TLC to contain predominantly monomeric GalA. TLC of the highly anionic, UV-absorbing fractions (16–18), which had co-electrophoresed with the ΔUA-GalA_*n*_, revealed predominantly the unsaturated dimer (ΔUA-GalA; the PL fingerprint). Interestingly, an additional product was detected by TLC in fractions 11–15: the putative RGL fingerprint product. One of these fractions (No. 12) was acid-hydrolysed, whereupon it yielded GalA and Rha (Fig. 4C), compatible with the proposed structure: ΔUA-Rha-GalA-Rha (monomeric ΔUA being largely lost in hot acid).

### RGL action in diverse ripe fruits

**Fig. 5.**
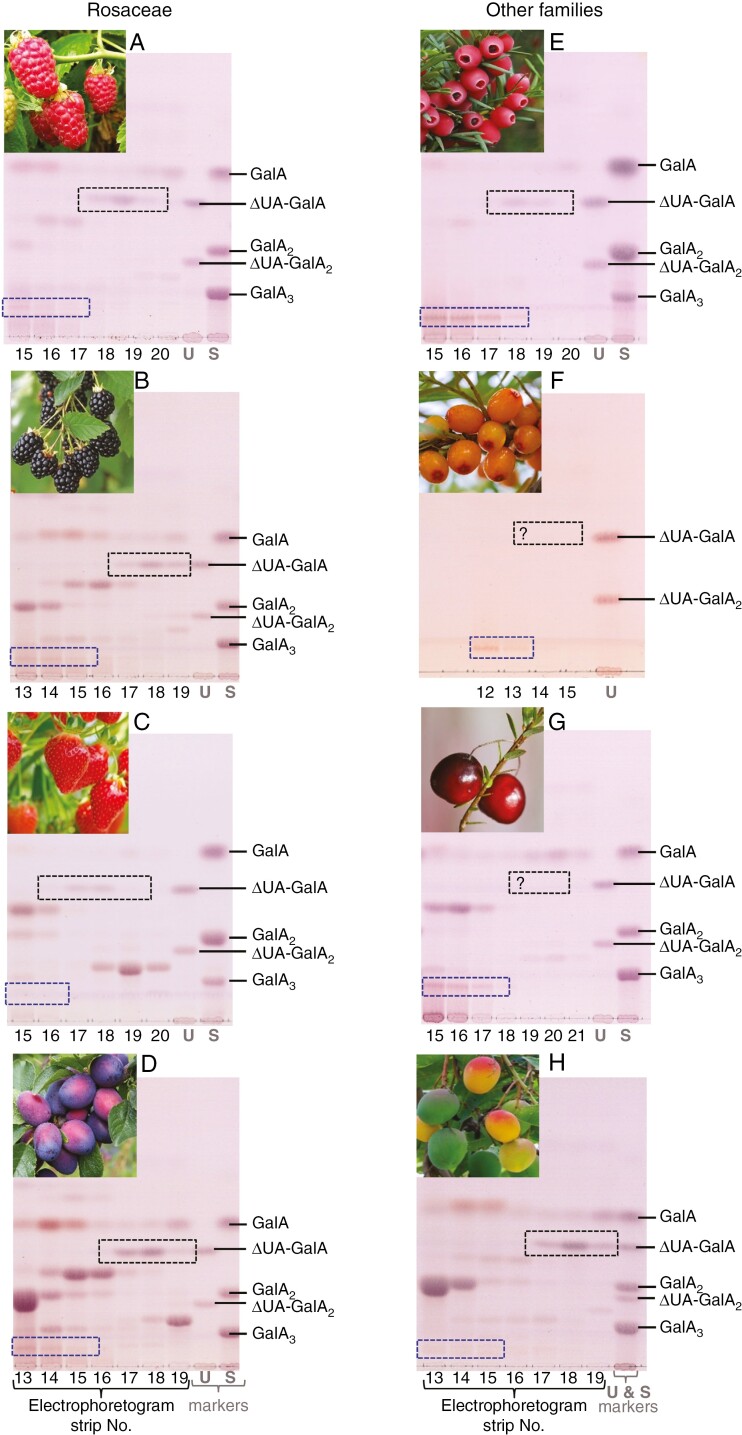
Detecting pectate lyase and RG lyase fingerprint products in Driselase digests of ripe fruit cell walls of various species. AIRs from fruits (or arils) of various plant species were Driselase-digested and analysed as in Fig. 4. The electrophoretogram fractions expected to contain unsaturated oligosaccharides were then subjected to TLC. The images represent only the zones containing the PL and RGL products. The PL- and RGL-fingerprints are in black and blue dashed rectangles respectively. Species tested were: A–D, Rosaceae (A, raspberry; B, blackberry; C, strawberry; D, plum); E–H, other families (E, yew; F, sea buckthorn; G, cranberry; H, mango). Markers: U, unsaturated oligogalacturonides; S, saturated oligogalacturonides, prepared as described by Al-Hinai *et al.* (2021).

**Fig. 6.**
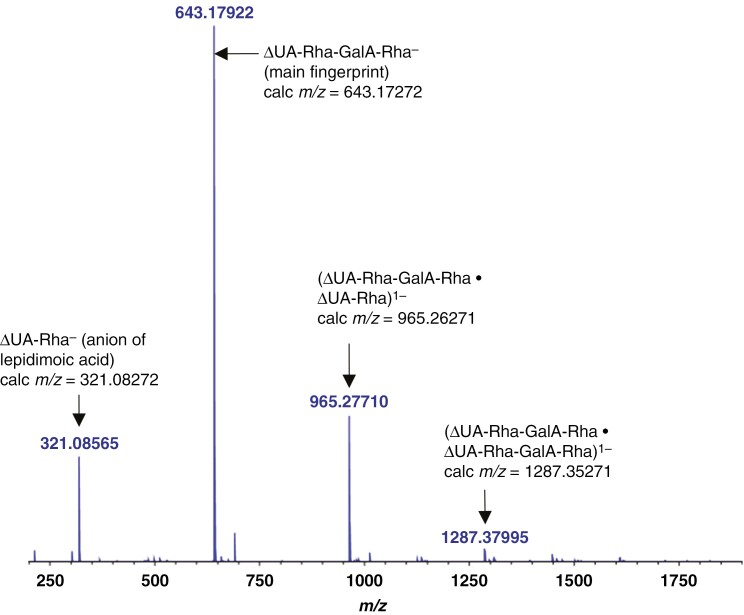
Negative-mode mass spectrometry of putative ΔUA-Rha-GalA-Rha obtained by Driselase digestion of de-esterified ripe apple fruit cell walls. Experimental negative-mode ESI FT–ICR mass spectrum of the fingerprint compound. Observed *m*/*z* values are labelled in blue; proposed identities and calculated *m*/*z* values are in black.

Using the methods described above for rowan berries, we detected the RGL fingerprint product (ΔUA-Rha-GalA-Rha) in Driselase digests of cell walls from ripe fruits of several other species including yew (arils), sea buckthorn, cranberry, raspberry, blackberry, strawberry, plum and mango (Fig. 5, where the fingerprint compound is marked with a blue rectangle). In addition, the results of similar experiments were reported previously for pear, apple and date (figs 6a, 8 and S1 of Al-Hinai *et al.*, 2021), where the compound now identified as ΔUA-Rha-GalA-Rha was labelled ‘X’. The detection of ΔUA-Rha-GalA-Rha in diverse fruit cell-wall digests indicates the pre-harvest *in-vivo* action of endogenous RGL.

Additional Driselase products are visible on TLCs of several species (Fig. 5). These products were not identified. Furthermore, several species generated a line of monomeric GalA spots in electrophoresis fractions 12–21: it is possible that these spots arose from unidentified GalA-based products that migrated rapidly on electrophoresis but were unstable when the electrophoretogram was dried and/or the compounds were eluted from the paper and loaded on the TLC plate.

### Mass spectrometric confirmation of the identity of the in-vivo RGL action product: apple as an example

The putative RGL fingerprint product, eluted from preparative TLC of the Driselase digest of apple AIR, was analysed by negative-mode electrospray-ionization FT-ICR MS. This gave a prominent peak with *m/z* 643.17 922 (Fig. 6), which is within 10 ppm of the 643.172 717 calculated for the (ΔUA-Rha-GalA-Rha)^1−^ anion, (C_24_H_35_O_20_)^1−^.

Additional peaks corresponding to a disaccharide, a hexasaccharide and an octasaccharide, [ΔUA-Rha-(GalA-Rha)_*n*_], where *n* is 0, 2 or 3, were also detected (Fig. 6). The disaccharide, ΔUA-Rha (peak at 321.08 565; theoretically 321.08 272), is known as lepidimoic acid (Hasegawa *et al.*, 1992; Iqbal *et al.*, 2016) and is suggested to be a break-down product of the predominant tetramer. The ‘octasaccharide’, on the other hand, could be either (a) a singly charged octasaccharide (ΔUA-Rha-GalA-Rha-GalA-Rha-GalA-Rha)^1–^ carrying one ΔUA residue or (b) a singly charged non-covalent adduct of two tetrasaccharides, each with one ΔUA residue, (ΔUA-Rha-GalA-Rha •ΔUA-Rha-GalA-Rha)^1–^. Although (a) and (b) would have identical *m*/*z* values, we prefer the latter interpretation: since the compound analysed in Fig. 6 was obtained from a single TLC band, it would not be expected to contain species ranging from the tetrasaccharide to the octasaccharide. A similar adduct (dimer•tetramer) is suggested for the observed ‘hexasaccharide’.

### Quantification of PL and RGL products in a Driselase digest: date as an example

The number of lyase-catalysed cleavage events per unit length of a polysaccharide chain can be estimated by the ratio of the ΔUA-containing ‘fingerprint’ compound to the total quantity of the substrate polysaccharide. For example, the number of RGL cleavage events per unit length of RG-I backbone is given by the yield of the tetrasaccharide ΔUA-Rha-GalA-Rha relative to the total RG-I-derived GalA + Rha monosaccharides in the Driselase digest of a fruit cell wall. Likewise, the number of PL cleavage events per unit length of HG can be calculated as the ratio of ΔUA-GalA to total HG-derived GalA monosaccharides in the Driselase digest (as in Al-Hinai *et al.*, 2021). For quantification of the Driselase products on thymol-stained TLCs, we needed to know:

(a) The amount of thymol-generated colour obtained per nmol of each of the residues (ΔUA, GalA and Rha) that constitute the ‘fingerprint’ oligosaccharides. An oligosaccharide gives a colour yield equal to the sum of its constituent monosaccharide residues because, when the TLC plate is heated at 105 °C in thymol/H_2_SO_4_, the oligosaccharide is cleaved into monomers which then each stain independently.
(b) Quantification of total HG and the total RG-I backbone in the fruit AIR. Driselase hydrolyses HG completely to the monosaccharide GalA, and hydrolyses the backbone of RG-I to GalA + Rha. It does not hydrolyse RG-II (Chormova *et al.*, 2014). RG-I has a GalA : Rha ratio of ~1 : 1 (mol/mol) (Yapo, 2011; Kaczmarska *et al.*, 2022). Therefore, in a Driselase digest of total cell walls, GalA minus Rha equals the total amount of HG backbone, and 2 × Rha equals the total amount of RG-I backbone. The Ara/Gal-rich side-chains of RG-I are also hydrolysed by Driselase to yield the corresponding neutral monosaccharides, but these do not need to be considered.

First we determined the thymol-generated colour yields of known amounts of GalA and Rha after TLC of a dilution series (Supplementary Data Fig. S2) and hence the colour yield for 1 nmol of these monosaccharides (GalA and Rha columns in Table S1).

Second we estimated the colour yield of 1 nmol ΔUA residue by reference to the staining intensity of ΔUA-GalA compared with that of equimolar ΔUA-GalO (where GalO is l-galactonic acid, the product formed when d-GalA is reduced by NaBH_4_) (Supplementary Data Fig. S3). In ΔUA-GalA, thymol generates colour from both the ΔUA and the GalA, whereas in ΔUA-GalO the colour arises only from the ΔUA residue because GalO (a non-reducing aldonic acid) does not react with thymol. As expected, ΔUA-GalA gave a colour yield higher than equimolar ΔUA-GalO (Table S1). The values in Table S1 allow TLC quantification of saturated residues (GalA, Rha) and the unsaturated products of the two lyases (the disaccharide ΔUA-GalA and the tetrasaccharide ΔUA-Rha-GalA-Rha).

For estimating RG-I and its RGL-generated non-reducing termini in Driselase digests of ripe date AIR (fig. 6A of Al-Hinai *et al.*, 2021), we measured the ImageJ ‘intensity density’ of the Rha and ΔUA-Rha-GalA-Rha spots in the digests (Supplementary Data Fig. S4; Table 1; raw data in Table S2), using conversion factors from Fig. S2 and Table S1. The RGL-generated termini comprised 1.34 % of the total RG-I backbone residues, half of which are Rha and thus potential cleavage sites for RGL. Thus, about one Rha residue (of RG-I) in every 37 had been cleaved by RGL in the date fruits analysed.

**Table 1.** Quantification of PL and RGL cleavage events in ripe date fruit detected by thymol staining of their fingerprint products. Based on fig. 6A of Al-Hinai *et al*. (2021). Date AIR (25 mg) was digested in Driselase and electrophoresed. Fractions were further analysed by TLC and the thymol-stained spots of ΔUA-GalA, GalA, ΔUA-Rha-GalA-Rha and Rha were identified. The spots were measured for ‘intensity density’ by ImageJ software and converted to nmol via the factors given in Supplementary Data Table S1 and Fig. S2. From the data (raw data are given in Table S2), the polysaccharide components listed below were calculated.

Residues (nmol) of:	Total nmol of fingerprint compound	Cleavage events per 100 backbone residues
PL-generated termini (nmol ΔUA-GalA)	1.70	0.75 (HG backbone)
Homogalacturonan backbone (nmol free GalA minus nmol free Rha)	224	
RGL-generated termini (nmol ΔUA-Rha-GalA-Rha)	0.26	1.34 (RG-I backbone)
RG-I backbone (2 × nmol free Rha)	18.6	

Comparable analysis of the ΔUA-GalA (disaccharide) product relative to the backbone of homogalacturonan indicates that ~0.75 % of the GalA residues of HG had been cleaved by PL.

## DISCUSSION

Acting on RG-I, RGL cleaves the α-(1→4)-glycosidic bond between Rha and GalA by β-elimination, creating a double bond between C-4 and C-5 in the GalA residue (making the unsaturated residue, ∆UA) at the new non-reducing terminus [Fig. 1 reaction (i)]. Following partial digestion of commercial potato RG-I by commercial RGL, Driselase was able to release a tetrasaccharide (ΔUA-Rha-GalA-Rha), which was detected on TLC (Fig. 2) and its constitution confirmed by MS (Fig. 3; *m*/*z* of the Na^+^ adduct 667.175). The same structure was also produced by a fungal RGL acting to completion on RG-I (Azadi *et al.*, 1995; Naran *et al.*, 2007). A structure with the same backbone as above was also found after complete digestion of RG-I by an *Aspergillus* RGL (Mutter *et al.*, 1996) although in that case the two Rha moieties each carried a neutral β-Gal residue as a side-chain, making a hexasaccharide. We also confirmed by TLC (Fig. 2B; Supplementary Data Fig. S1) and by MS that Driselase lacks RGL activity, supporting its suitability for releasing RGL products from fruit AIR.

The ‘fingerprinting’ strategy devised here is largely based on the strong acidity (low *p*K_a_) of the ΔUA residue compared with the weaker acidity of a saturated GalA residue and consequent difference in electrophoretic mobility in an acidic buffer (pH 2.0). Kohn and Kováč (1978) reported *p*K_a_ values of 3.51 and 3.10 for GalA methyl glycoside and ΔUA methyl glycoside respectively, these glycosides modelling the GalA and ΔUA residues present in ‘fingerprint’ oligosaccharides.

The RGL action fingerprint (ΔUA-Rha-GalA-Rha) was also obtained by Driselase digestion of ripe fruit AIR and documented by electrophoresis and TLC (Fig. 4A, B). RGL products overlapped with PL products on paper electrophoresis (Fig. 4A), but were resolved clearly on TLC (Fig. 4B) as they have a slightly lower mobility on electrophoresis than PL products and run much slower on TLC owing to their larger size. The mass of the ΔUA-Rha-GalA-Rha isolated from apple fruits was confirmed by MS (Fig. 6) and found to match that of the product obtained *in vitro* by digestion of commercial RG-I by commercial RGL (Fig. 3). Furthermore, TLC analysis of the acid hydrolysis products of the ‘fingerprint’ (Fig. 4C) supported its identity as ΔUA-Rha-GalA-Rha.

The RGL action products were also successfully detected in ripe rowan berry, apple, pear, raspberry, blackberry and plum (all dicots of the family Rosaceae), as well as mango (Anacardiaceae) and yew (a gymnosperm) in addition to cranberry (Ericaceae) and sea buckthorn (Elaeagnaceae) where PL action products were undetectable.

It might be speculated that some of the detected ΔUA residues arose by RGL action *post mortem*, while the AIR was being washed, rather than *in vivo*. However, the AIR was prepared in 75 % ethanol containing 5 % formic acid, which blocks RGL activity, so we can conclude that the ΔUA residues were generated *in vivo*.

The yields of the tetrasaccharide fingerprint of RGL action differed between the ripe fruit of various species: apple > pear > yew > date > rowan > cranberry > plum > sea buckthorn > blackberry > raspberry > mango [semi-quantified from scans of TLC plates shown in Fig. 5 and the band labelled ‘X’ in Al-Hinai *et al.* (2021)]. The low yield in, for example, mango (Fig. 5) supports the idea that the tetrasaccharide does not arise from Driselase autolysis or the presence of RGL activity in Driselase. The yield of the fingerprint from aril cell walls of yew (a gymnosperm) supports the widespread taxonomic distribution of RGL action *in vivo*.

The estimated degree of RG-I cleavage by RGL in living fruits disregards the possibility that Rha residues carrying Ara and/or Gal side-chains may have been resistant to RGL. It is not yet known whether plant RGLs can, like *Aspergillus* RGL (Mutter *et al.*, 1996), cleave the RG-I backbone adjacent to the Ara/Gal-rich side-chains. Furthermore, the degree of substitution of RG-I with such side-chains varies between plant species (Kaczmarska *et al.*, 2022). This is a topic for future investigation.

In the cell walls of ripe dates, there were 6.5× more total PL products than RGL products (Table 1). Nevertheless, these walls contain 12× more homogalacturonan than RG-I. Therefore, the yield of RGL products compared with PL products (expressed as cleavage events per 100 backbone residues) suggests that rhamnogalacturonan had been more extensively lyase-digested *in vivo* than had homogalacturonan.

Thus, this work supports a significant role for RGL in the softening of living fruit during ripening. Future work will be necessary to rank the relative contributions of endo-hydrolases (EPG and possible RGase), endo-lyases (RGL and PL) and reactive oxygen species in bringing about the degradation of fruit pectin *in vivo* as well as evaluating the synergistic effects of other pectin-modifying enzyme activities such as exo-polygalacturonase, pectin methylesterase, pectin acetylesterase, β-d-galactosidase and α-l-arabinosidase.

## CONCLUSIONS

The natural softening of fruit mesocarp is due to mechanical changes occurring in primary cell walls and middle lamellae. These physical changes hinge on biochemical modification of wall-matrix and middle-lamellar polysaccharides. We currently have an incomplete view of the biochemical processes involved and thus of the most auspicious targets by which to control softening. There is an extensive literature on gene expression during fruit ripening. There is less information on the biochemistry of the encoded enzymes (activity and substrate specificity), and still less on the enzymes’ relative *in-vivo* action as revealed through studies of polysaccharide chemistry. Thus, although there are numerous suggestions as to the key processes affecting fruit softening, placing these in context and assessing their relative contributions remains an important challenge. The current paper is a step towards providing such knowledge, presenting a methodology to explore fruit softening from the ‘polysaccharide end’, complementing data obtained from the ‘transcriptome end’. Our results provide the first demonstration that RGL, previously explored in studies of fruit gene expression, proteomics and assaying *in-vitro* enzyme activity, exhibits *action* in the cell walls of soft fruits and may thus credibly be proposed to contribute to fruit softening. RGL catalyses a reaction which chemically resembles that of PL. However, their respective substrates are biosynthetically unrelated to each other: the pectic RG-I and homogalacturonan domains are produced by different sets of polysaccharide synthases. Therefore, RGL and PL can act independently of each other, so either or both can potentially govern the softening of the fruit cell wall and middle lamella. This paper therefore adds to our knowledge of the chemical basis of fruit softening – potentially offering novel strategies for manipulating the shelf-life of soft fruits. The methodology developed for monitoring RGL action (and, in a related paper, PL action; Al-Hinai *et al.*, 2021) provides the means to monitor the *in-vivo* cleavage of RG-I and (independently) homogalacturonan in response to any chosen means of changing these two activities – including site-directed mutagenesis, ‘genetic engineering’, and the treatment of fruit crops in the field, during transport and in post-harvest storage.

## SUPPLEMENTARY DATA

Supplementary data are available at *Annals of Botany* online and consist of the following.

Fig. S1. Ability of Driselase to digest various acidic plant cell-wall components. Fig. S2. Colour yields of GalA and Rha on a thymol-stained TLC plate. Fig. S3. Determining the thymol colour yield of ΔUA residues. Fig. S4. Zones selected from a ‘fingerprint’ TLC of date fruit for quantification of relevant products. Table S1. Quantitative ImageJ ‘intensity density’ of thymol-stained TLC spots. Table S2. Calculation of data for Table 1.

mcad197_suppl_Supplementary_Figures_S1-S4

mcad197_suppl_Supplementary_Tables_S1-S2
