# Supplementary material for: Fruit softening: evidence for rhamnogalacturonan lyase action in vivo in ripe fruit cell walls
Source: Ann Bot. 2024 Jan 5;133(4):547–58. doi: 10.1093/aob/mcad197 (PMC11037484; doi:10.1093/aob/mcad197)
Supplement: mcad197_suppl_Supplementary_Figures_S1-S4 [file mcad197_suppl_supplementary_figures_s1-s4.zip › mcad197_suppl_Supplementary_Figures_S1-S4.pptx]

## Slide 1
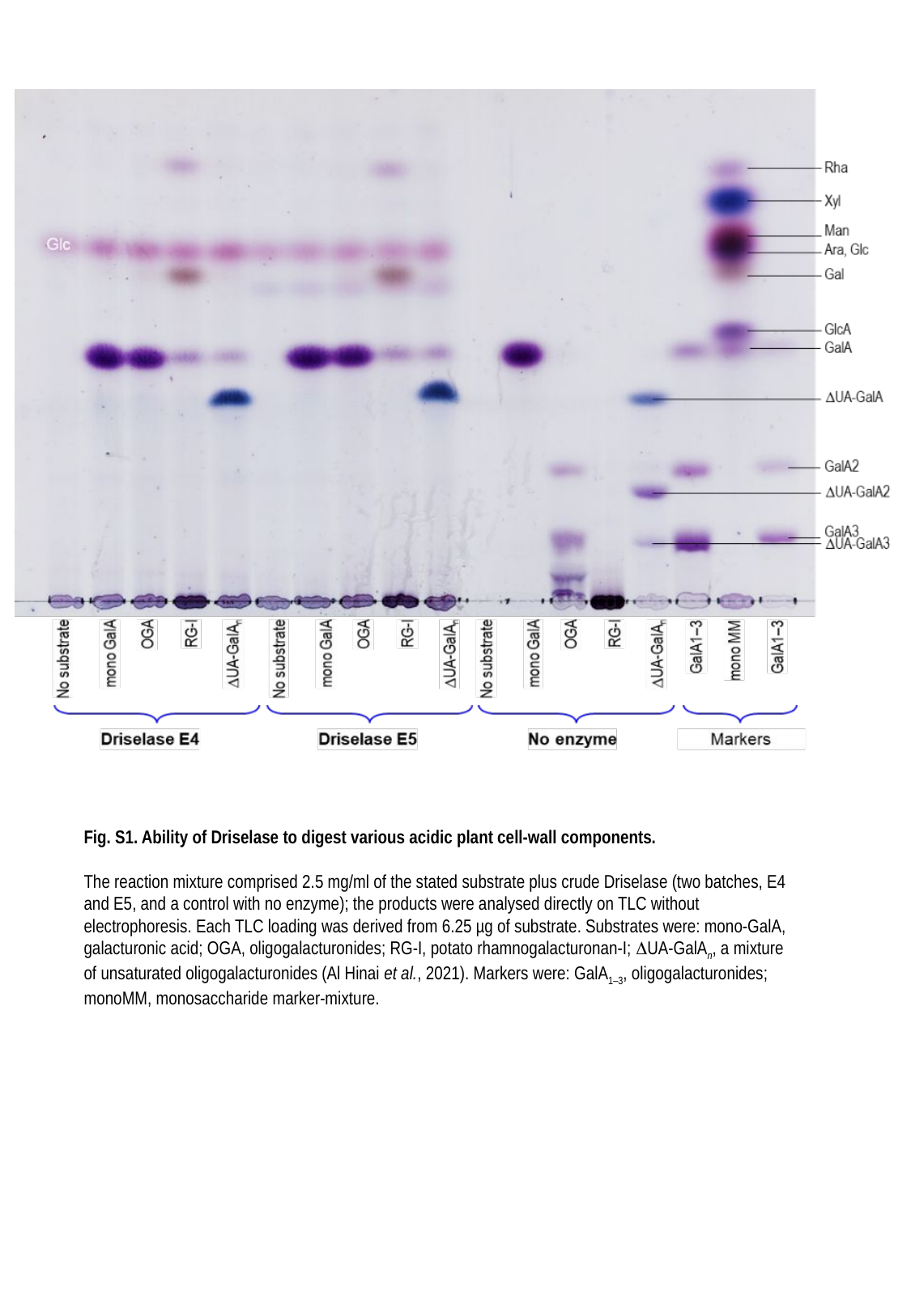

Fig. S1. Ability of Driselase to digest various acidic plant cell-wall components.
The reaction mixture comprised 2.5 mg/ml of the stated substrate plus crude Driselase (two batches, E4 and E5, and a control with no enzyme); the products were analysed directly on TLC without electrophoresis. Each TLC loading was derived from 6.25 µg of substrate. Substrates were: mono-GalA, galacturonic acid; OGA, oligogalacturonides; RG-I, potato rhamnogalacturonan-I; ΔUA-GalAn, a mixture of unsaturated oligogalacturonides (Al Hinai et al., 2021). Markers were: GalA1–3, oligogalacturonides; monoMM, monosaccharide marker-mixture.

## Slide 2
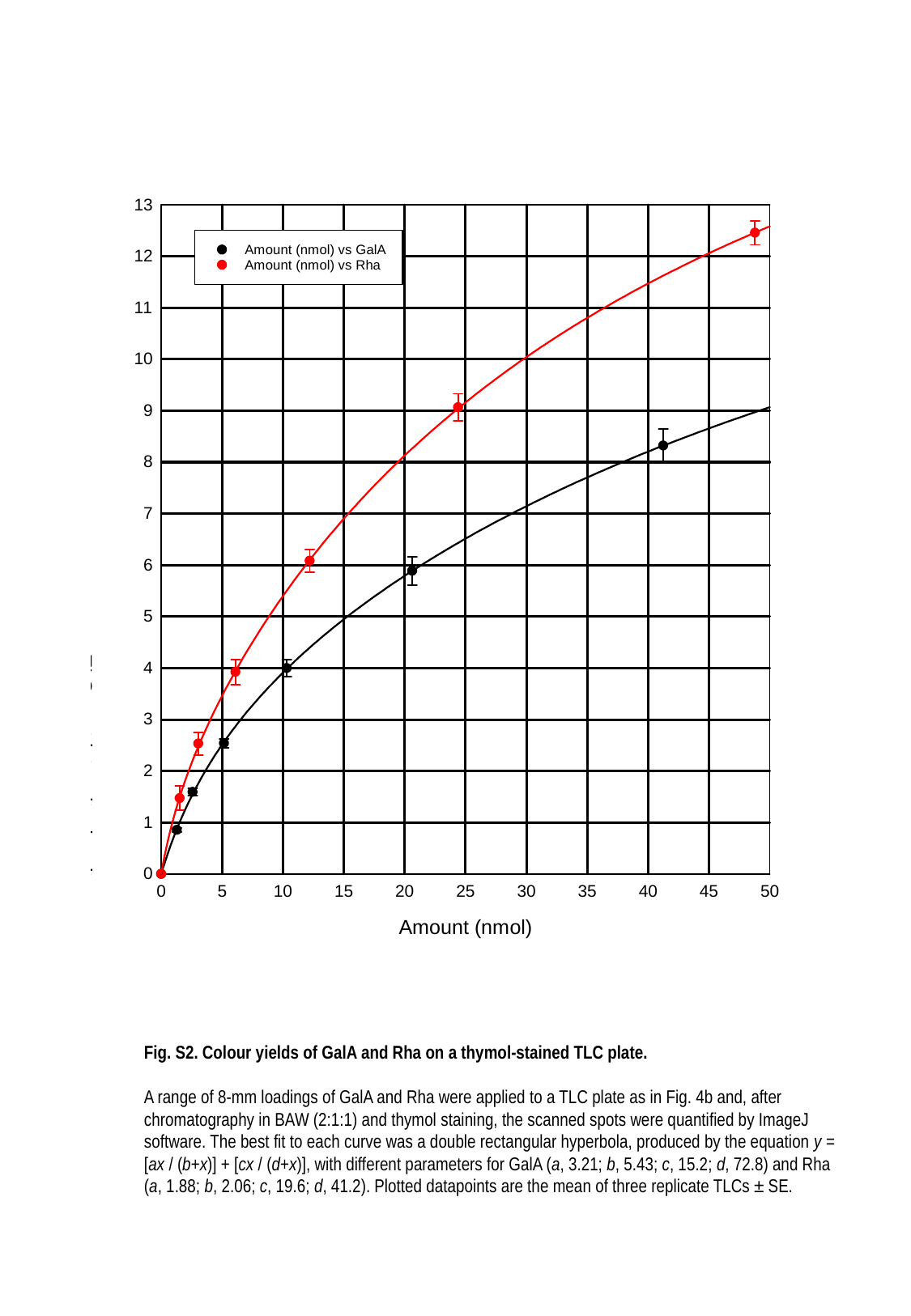

Fig. S2. Colour yields of GalA and Rha on a thymol-stained TLC plate.
A range of 8-mm loadings of GalA and Rha were applied to a TLC plate as in Fig. 4b and, after chromatography in BAW (2:1:1) and thymol staining, the scanned spots were quantified by ImageJ software. The best fit to each curve was a double rectangular hyperbola, produced by the equation y = [ax / (b+x)] + [cx / (d+x)], with different parameters for GalA (a, 3.21; b, 5.43; c, 15.2; d, 72.8) and Rha (a, 1.88; b, 2.06; c, 19.6; d, 41.2). Plotted datapoints are the mean of three replicate TLCs ± SE.

## Slide 3
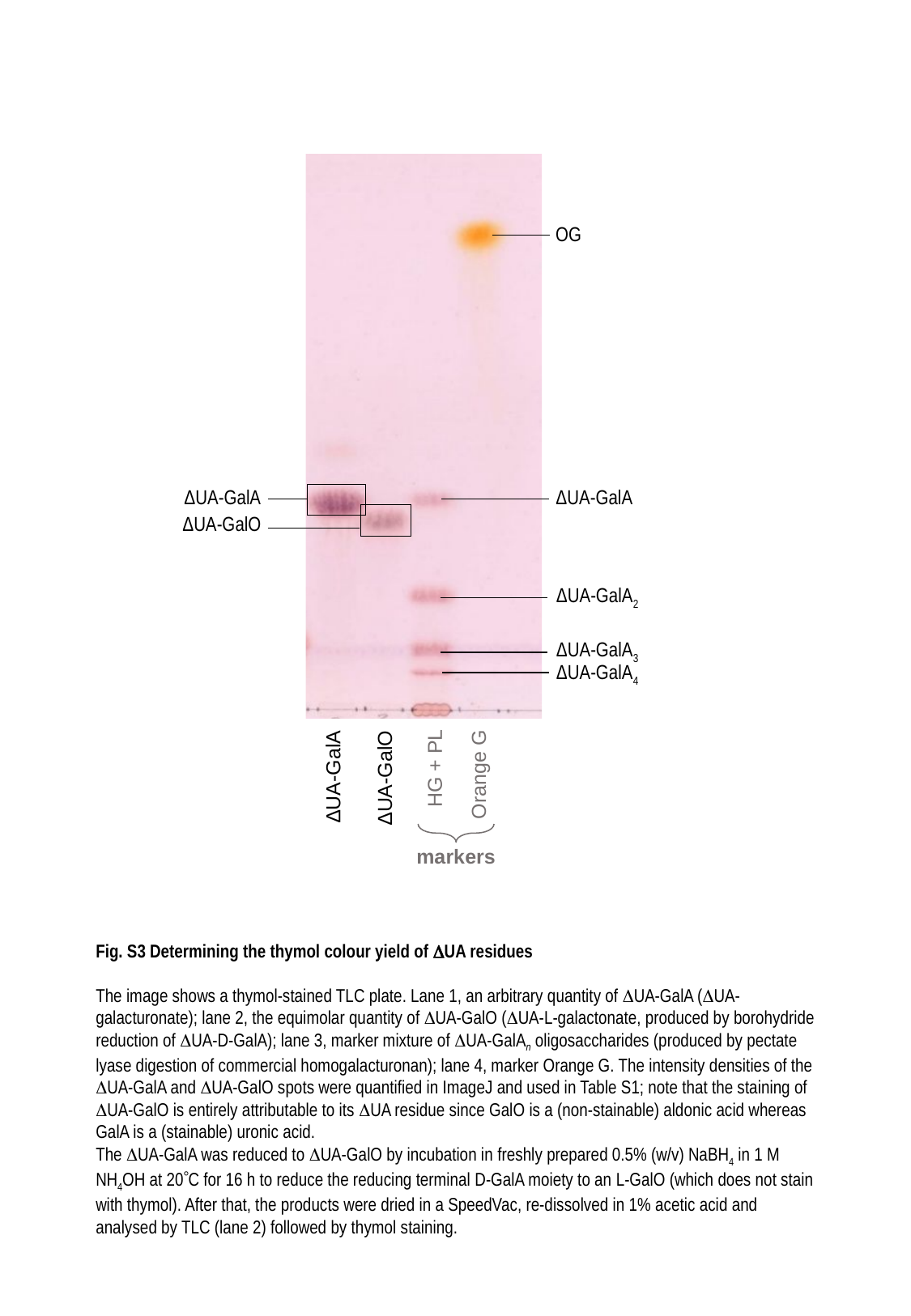

OG
ΔUA-GalA
ΔUA-GalA
ΔUA-GalO
ΔUA-GalA2
ΔUA-GalA3
ΔUA-GalA4
HG + PL
Orange G
∆UA-GalA
∆UA-GalO
markers
Fig. S3 Determining the thymol colour yield of ΔUA residues
The image shows a thymol-stained TLC plate. Lane 1, an arbitrary quantity of ΔUA-GalA (ΔUA-galacturonate); lane 2, the equimolar quantity of ΔUA-GalO (ΔUA-l-galactonate, produced by borohydride reduction of ΔUA-d-GalA); lane 3, marker mixture of ΔUA-GalAn oligosaccharides (produced by pectate lyase digestion of commercial homogalacturonan); lane 4, marker Orange G. The intensity densities of the ΔUA-GalA and ΔUA-GalO spots were quantified in ImageJ and used in Table S1; note that the staining of ΔUA-GalO is entirely attributable to its ΔUA residue since GalO is a (non-stainable) aldonic acid whereas GalA is a (stainable) uronic acid.
The ΔUA-GalA was reduced to ΔUA-GalO by incubation in freshly prepared 0.5% (w/v) NaBH4 in 1 M NH4OH at 20°C for 16 h to reduce the reducing terminal d-GalA moiety to an l-GalO (which does not stain with thymol). After that, the products were dried in a SpeedVac, re-dissolved in 1% acetic acid and analysed by TLC (lane 2) followed by thymol staining.

## Slide 4
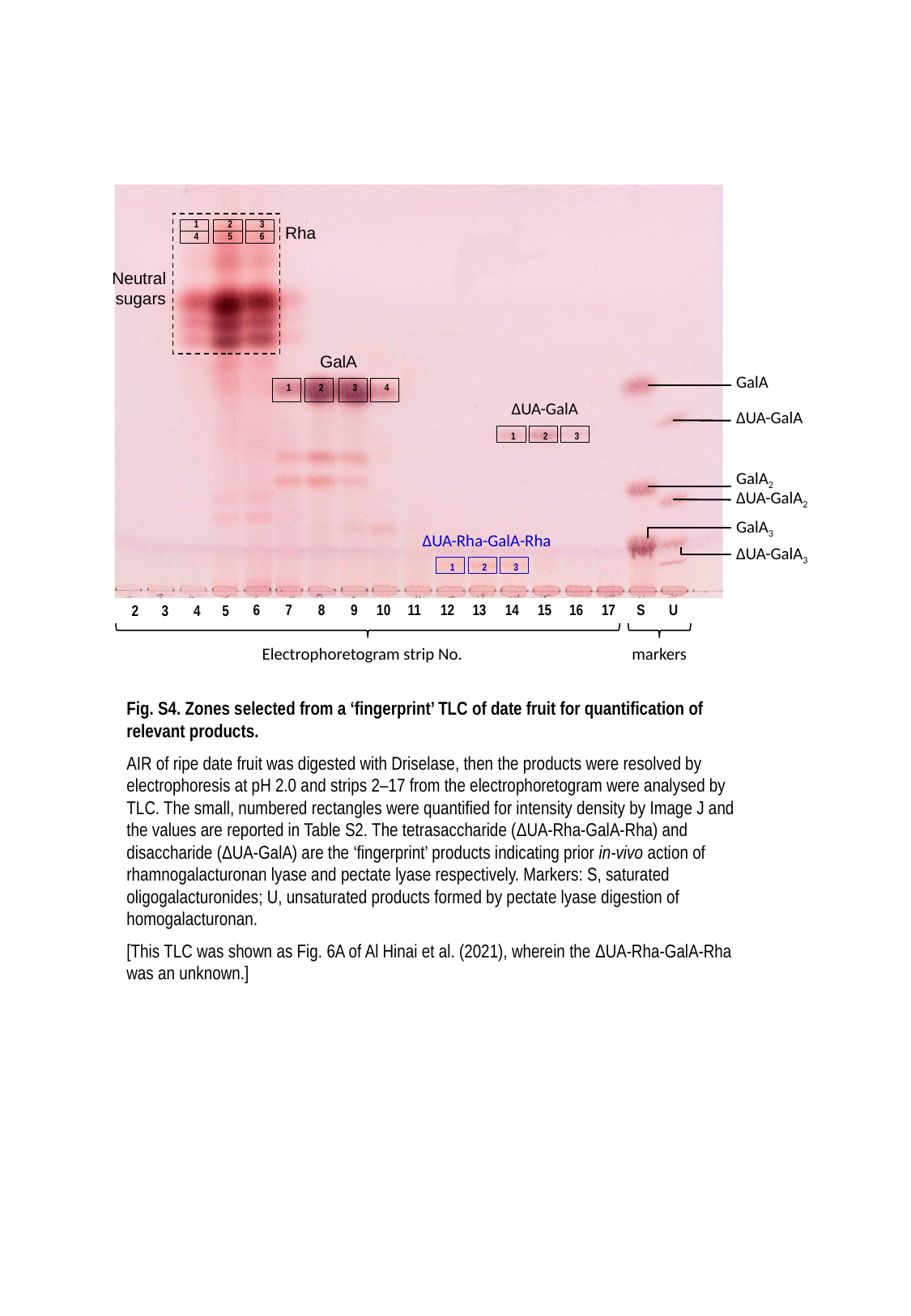

Rha
1
2
3
4
5
6
Neutral
sugars
GalA
GalA
1
2
3
4
ΔUA-GalA
ΔUA-GalA
1
2
3
GalA2
ΔUA-GalA2
GalA3
ΔUA-Rha-GalA-Rha
ΔUA-GalA3
1
2
3
6
7
8
9
10
11
12
13
14
15
16
17
S
U
2
3
4
5
Electrophoretogram strip No.
markers
Fig. S4. Zones selected from a ‘fingerprint’ TLC of date fruit for quantification of relevant products.
AIR of ripe date fruit was digested with Driselase, then the products were resolved by electrophoresis at pH 2.0 and strips 2–17 from the electrophoretogram were analysed by TLC. The small, numbered rectangles were quantified for intensity density by Image J and the values are reported in Table S2. The tetrasaccharide (ΔUA-Rha-GalA-Rha) and disaccharide (ΔUA-GalA) are the ‘fingerprint’ products indicating prior in-vivo action of rhamnogalacturonan lyase and pectate lyase respectively. Markers: S, saturated oligogalacturonides; U, unsaturated products formed by pectate lyase digestion of homogalacturonan.
[This TLC was shown as Fig. 6A of Al Hinai et al. (2021), wherein the ΔUA-Rha-GalA-Rha was an unknown.]
