# Supplementary material for: Fruit softening: evidence for rhamnogalacturonan lyase action in vivo in ripe fruit cell walls
Source: Ann Bot. 2024 Jan 5;133(4):547–58. doi: 10.1093/aob/mcad197 (PMC11037484; doi:10.1093/aob/mcad197)
Supplement: mcad197_suppl_Supplementary_Tables_S1-S2 [file mcad197_suppl_supplementary_tables_s1-s2.docx]

**Table S1**. Quantitative ImageJ ‘intensity density’ of thymol-stained TLC spots

| Parameter | GalA | ΔUA | ΔUA-GalA | Rha | ΔUA-Rha-GalA-Rha |
| --- | --- | --- | --- | --- | --- |
| Relative molecular mass | 194 | 176 | 352 | 164 | 644 |
| Intensity density for 1 nmol on TLC, from standard curve (Fig. S2) | **0.705** (a) |  |  | **1.082** (b) |  |
| Intensity density for 1 nmol, calculated using ‘d’ as in footnote.* |  | 0.408 (c)  (= a / d) |  |  |  |
| Intensity density for 1 nmol of oligosaccharide, calculated by summing its residues. |  |  | **1.113**  (= a + c) |  | **3.277**  (= a + 2b + c) |

*Calculation of the relative colour yield of thymol-stained ΔUA residues:

Observed ImageJ ‘intensity density’ of the TLC spot of an arbitrary amount of ΔUA-Gal**A** (Fig. S3, lane 1) = 5.974, which is the colour yield that would have been given by 21.19 nmol of pure GalA (from Fig. S2).

Observed intensity density of the same arbitrary (molar) amount of ΔUA-Gal**O** (Fig. S3, lane 2) = 3.351, which is entirely due to the ΔUA moiety and is equivalent to 7.77 nmol GalA (from Fig. S2).

The 21.19 nmol (GalA equiv) of ΔUA-GalA clearly contains the same quantity of ΔUA as equimolar ΔUA-GalO (7.77 nmol GalA equiv), so the remainder of the staining (21.19 − 7.77 = 13.42 nmol GalA equiv) must have been due to 13.42 true nmol of GalA.

Hence 7.77 ‘nmol GalA equiv’ of ΔUA implies 13.42 true nmol of ΔUA, so thymol is 13.42/7.77 = 1.727 times more effective at detecting GalA residues than it is at detecting ΔUA residues. The value 1.727 is used as ‘d’ in the Table above.

Note: GalA is a thymol-stainable uronic acid, whereas GalO is a non-stainable aldonic acid formed from GalA by reduction with NaBH_4_.

**Table S2: Calculation of data for Table 1**

**(a) Raw data**

|  | **Free GalA** | | **Free Rha** | | **Disaccharide**  **(ΔUA-GalA)** | | | **Tetrasaccharide**  **(ΔUA-Rha-GalA-Rha)** | | |
| --- | --- | --- | --- | --- | --- | --- | --- | --- | --- | --- |
|  | Measur­ed int’y density | Calcul­ated nmol | Measur­ed int’y density | Calcul­ated nmol | Measur­ed int’y density | nmol calculated as if GalA | Corrected* nmol ΔUA-Gal | Measur­ed int’y density | nmol calculated as if GalA | Corrected* nmol tetrasacch |
| *Rectangle 1* | 5.66 | 19.2 | 1.11 | 1.03 | 0.469 | 0.637 | 0.40 | 0.296 | 0.390 | 0.084 |
| *Rectangle 2* | 11.7 | 98.0 | 1.94 | 2.15 | 1.23 | 1.93 | 1.22 | 0.391 | 0.524 | 0.113 |
| *Rectangle 3* | 11.8 | 98.3 | 2.09 | 2.39 | 0.094 | 0.12 | 0.08 | 0.239 | 0.312 | 0.067 |
| *Rectangle 4* | 5.47 | 18.0 | 0.991 | 0.897 |  |  |  |  |  |  |
| *Rectangle 5* |  |  | 1.46 | 1.46 |  |  |  |  |  |  |
| *Rectangle 6* |  |  | 1.40 | 1.39 |  |  |  |  |  |  |
| ***Total*** |  | **233** |  | **9.31** |  |  | **1.70** |  |  | **0.26** |
|  |  | **(A)** |  | **(B)** |  |  | **(C)** |  |  | **(D)** |

Intensity densities of thymol-stained TLC spots (each in the form of 3–6 individual rectangles) were measured by ImageJ, then converted to nmol as described in Table S1 and Fig. S2.

*Corrected by reference to constants in Table S1.

**(b) Lyase-generated fingerprint yields compared with polysaccharide backbone residues**

| **Component** | **nmol in digest** | **Calculated as** [see raw data, above] |
| --- | --- | --- |
| ***Backbone residues*** |  |  |
| Homogalacturonan backbone | **224** | (A) – (B) |
| RG-I backbone (Rha & equimolar GalA) | **18.6** | 2 × (B) |
| ***Lyase-generated termini*** |  |  |
| PL cleavage products | **1.70** | (C) |
| RGL cleavage products | **0.26** | (D) |
